# Does disease incite a stronger moral appeal than health?

**DOI:** 10.1186/s12916-023-03110-3

**Published:** 2023-11-06

**Authors:** Bjørn Hofmann

**Affiliations:** 1 Norwegian University of Science and Technology - Gjøvik Campus: Norges Teknisk-Naturvitenskapelige Universitet i Gjøvik, Gjøvik, Innlandet Norway; 2 Centre for medical ethics, Faculty of Medicine, University of Oslo, Oslo, Norway

**Keywords:** Health, Disease, Wellbeing, Suffering, Moral appeal, Duty, Imperative

## Abstract

Is disease demotion more important than health promotion? The question is crucial for the ethos of medicine and for priority setting in healthcare. When things get tough, where should our attention and resources go: to health or disease? This study investigates two general perspectives on health and disease to address whether there is a stronger moral appeal from people’s disease than from their health. While naturalist conceptions of health and disease are mute on moral appeal, normativist conceptions give diverse answers. Classical utilitarianism provides a symmetrical view of health and disease, according to which we have an equally strong moral appeal to further health as we have to reduce disease. Other normativist positions argue that there is an asymmetry between health and disease providing substantial support for a stronger moral appeal from disease than from health. This has a wide range of radical implications, especially within priority setting. In particular, treatment, palliation, and prevention of disease should have priority to the promotion and enhancement of health.

## Background

Is disease demotion more important than health promotion? That is, is there a stronger moral appeal from people’s disease than from their health? This question is pertinent as the possibilities to improving people’s health and handle their disease by far outruns the allocated resources. Healthcare expenditures amounted to 18% of the GDP in the USA and 12% of the GDP in the UK (in 2020) and the numbers have been increasing in many countries [1]. Moreover, it has been argued that we provide “too much care for healthy people, and not enough care for the sick” [2]. As we have limited resources, should we give higher priority to handling people’s disease than to promoting their health? Should we treat persons suffering from malaria or diabetes before we promote their health and happiness?

One way to address this question is to investigate whether there is a stronger moral appeal to demote people’s disease than to promote their health. To address this issue, the article will start by clarifying what is meant by moral appeal. Then it will investigate whether there is an asymmetry in moral appeal with respect to two major accounts of health and disease. First, it will investigate a general normativist wellbeing-based conception of health versus a suffering-based conception of disease. Thereafter, the article will investigate a general naturalist function-based conception of health and a dysfunction-based conception of disease.

Specifically, this study will focus on the moral appeal of the health and disease of one person to other persons in general and to health professionals, healthcare institutions, and states in particular. If a person is suffering from disease, there is a broadly shared intuition that we are obliged to help that person [3]. Do we have an corresponding duty to promote their health?

Most governmental entities have measures and institutions to demote (mitigate, relieve, treat) disease and to promote their populations’ health. Many countries also legally encode a general civil duty to help persons when they are harmed or when health or life itself is endangered — often called a “rule to rescue” [4]. This seems to be rooted in deep cultural taboos against abandoning people in dire straits. Additionally, health professionals are under an even stronger obligation to aid, which is also codified in the various countries’ health legislation.

Correspondingly, there is a generally shared intuition that we are obliged to promote other persons’ health. Health promotion has been on the agenda of international organizations such as the World Health Organization and national health authorities for decades, and it has long been claimed that “prevention is better than cure” [5] and that “an ounce of prevention is worth a pound of cure” [6]. While such adages have been challenged [7], they still stand strong. While crucial for priority setting, the issue of whether it is more important to demote disease than to promote health has gained surprisingly little attention.

Accordingly, this study will investigate the relationship between the intuitions that we are obliged to demote disease and promote health. To do so, it will investigate the moral appeal of health and disease to other individuals, professionals, institutions, and societies. However, first, we need to clarify what is meant by moral appeal.

## What does moral appeal mean?

Do health and/or disease represent a “moral imperative” [8, 9], a “moral duty” [10], a “moral pressure” [11], a “moral demand,” a “moral claim,” a “moral plea,” a “moral call” [12], a “moral affect,” or something else (such as recommendation, suggestions, exhortations)?

**Fig. 1:**
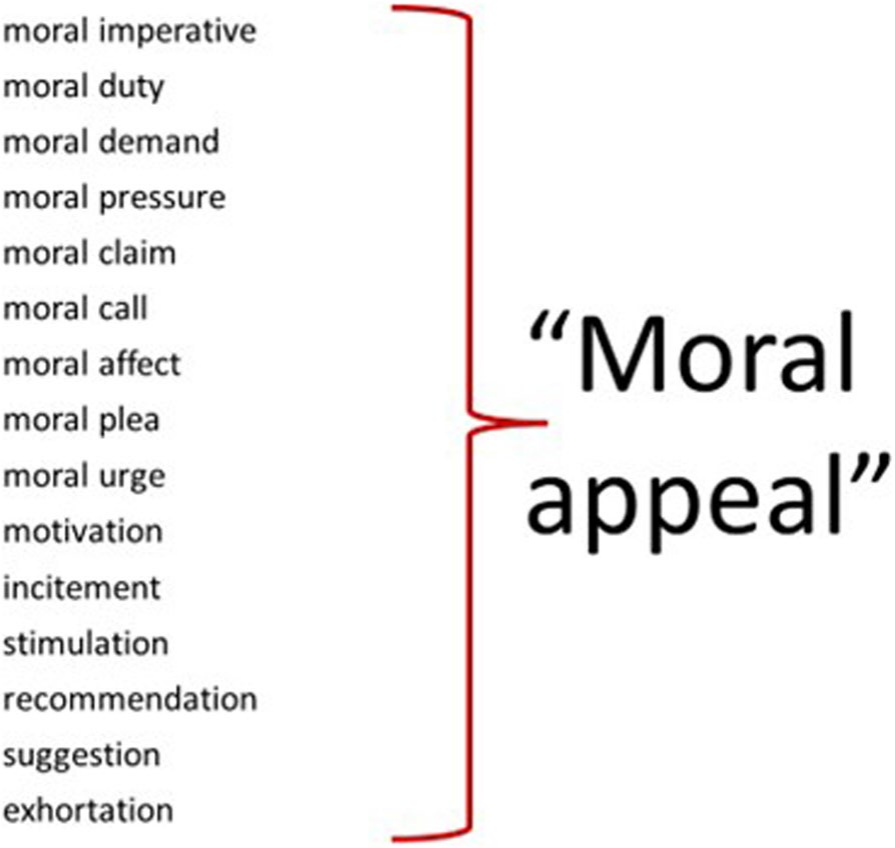
Various types of moral impetus and how “moral appeal” is used as a generic term to cover them

As this study investigates the general question of whether there is a difference in the moral appeal between health and disease, it will not be limited to any specific type or strength of moral impetus. “Moral appeal” will be used as a generic term to refer to any type of moral impetus as illustrated in Fig. 1.

Correspondingly, the strength of the moral appeal may depend on the strength of a person’s health (gain) and on the severity of disease. While I will briefly return to this topic at the end, the main issue of this article is the (a)symmetry with respect to the moral appeal from people’s health and disease in general.

The reason why the moral appeal from health and disease are so important in the healthcare setting is because health and disease are core concepts in the goals of medicine and healthcare, i.e., to cure or alleviate disease and promote health [13].

## Main text

### Two main conceptions of health and disease

There are many conceptions of health and disease [14–17], and the moral appeal may vary substantially with these conceptions. While it is beyond the scope of this study to investigate all the specific concepts of health and disease and their respective moral appeals, it will scrutinize two general conceptions, i.e., naturalist and normativist conceptions of health and disease.

According to a general naturalist conception, health and disease do not have any moral appeal, as they are value-neutral concepts. Normativists, on the other hand, claim that health and disease are value-laden concepts but they have diverting opinions on the (a)symmetry of their moral appeal. Table 1 provides an overview of the general conceptions of health and disease and the corresponding answers to the question of moral appeal.

**Table 1:** Summary of general normativist and naturalist conceptions of health and disease and the corresponding answer to the question of (a)symmetry in moral appeal

	**Naturalist**	**Normativist**
**Health and disease**	Health and disease are symmetric concepts (theoretical)	a) Symmetry (Utilitarianism)
		b) Asymmetry (several positions and perspectives)
**Moral appeal**	Health and disease have no (inherent) moral appeal	a) Health and disease have corresponding moral appeal (Utilitarianism)
		b) Disease has a stronger moral appeal than health (several positions)

How do the naturalists and normativists come to their conclusions?

#### Naturalists: symmetrical and value-neutral concepts without moral appeal

In a purely naturalistic (and theoretical) sense health and disease are symmetrical concepts, and there is no moral appeal from neither of them. For example, Christopher Boorse, who is one of the most renowned proponent of a naturalist conception of health and disease, argues for a conceptual symmetry between health and disease in terms of health being the absence of disease, and disease implying the absence of health. Health is normal functioning and disease is a type of internal state which impairs health, i.e., reduces one or more functional abilities below typical efficiency [18, 19].

Hence, health and disease are symmetrical concepts as the one is defined in terms of the absence of the other. However, they do not have any moral appeal, as they are value neutral.

It is important to notice that naturalists also can acknowledge practical value-laden conceptions, such as illness. For example, Boorse defines illness as an undesirable incapacitating disease: “being ill involves having a disease serious enough to be somewhat incapacitating, which thereby supports normative judgments about treatment and responsibility” [18]. Thus illness can have a moral appeal, but this brings us to the normativist camp to be discussed below.

In sum, a naturalistic account, like Boorse’s, holds symmetrical conceptions of health and disease, but the concepts do not have any moral appeal, as they are value neutral. Boorse has been extensively criticized for having implicitly value-laden conceptions of health and disease [16, 17, 20–25] and thereby implicitly having a normativistic account of health and disease.

This is certainly not the place to enter the interesting debate on various naturalistic conceptions of health and disease [26]. For the purpose of this study, it suffices to notice that naturalistic conceptions of health and disease do not provide any generic answers to the question of whether disease has a stronger moral appeal than health.

#### Normativists: value-laden and (a)symmetrical moral appeal

Many normativists define health in terms of wellbeing and disease in terms of suffering. WHO’s definition of health is one example of this, according to which health is “a state of complete physical, mental, and social wellbeing and not merely the absence of disease or infirmity” [27].

Accordingly, the difference in moral appeal between health and disease follows the difference in moral appeal between wellbeing and suffering. In the quite extensive literature on the relationship between wellbeing and suffering, there are two main positions, the symmetry view (dominated by classical utilitarianism) and the asymmetry view (with a wide range of philosophical underpinnings). A submitted review provides an overview of these positions and a summary is presented in Table 2.

**Table 2:** Difference in moral appeal from wellbeing and suffering

**View**	**Symmetry view**	**Asymmetry view**
**Description**	We have an equally strong moral obligation to promote other persons’ wellbeing as we have to reduce their suffering	We have stronger moral obligations towards other persons’ suffering than towards their wellbeing
**Theories, positions, or perspectives defending the view**	Classical utilitarianism	• Negative utilitarianism • Ontology: substantial differences, different kinds, including eliminativism • Value theory, axiology: there are relative differences, difference in strength • Rule-based ethics/meta-ethics: asymmetrical purpose of morals • Virtue ethics: the moral asymmetry of virtues • Philosophy of language: logical and conceptual differences • Phenomenology: differences in phenomena

Applying Mayerfeld’s definition on wellbeing and suffering [28, 29] for health and disease, the symmetry view would be that *we should maximize the total surplus of health over disease; therefore, it is always better (other things being equal) to bring about a larger increase in health than a smaller reduction of disease, and to bring about a greater reduction of less severe disease than a smaller reduction of more severe disease*.

While Classical utilitarianism supports this symmetry view [28, 29], a range of positions support an asymmetry view between health and disease, and thus a difference in moral appeal. Accordingly, one can use negative utilitarianism [30] to argue that there is a direct moral appeal from disease that is not matched by a moral appeal from health. The pain and suffering related to disease pose a moral appeal that does not correspond to a moral appeal related to the wellbeing of health. For each state of disease, there is not a matching state of health.

In line with arguments for an ontological distinction between wellbeing and suffering, it can be maintained that health and disease are substantially dissimilar, e.g., that they are different kinds [28, 29, 31]. Disease has morally relevant elements or features (such as pain and suffering) that health does not have. An extreme version of this view (eliminativism) would claim that one of the two (health or disease) does not exist, thus giving priority to the other. Gadamer’s conception of health as something ungraspable and enigmatic that is given and cannot be produced or “effected” [32] is but one example of this. If health is impossible or more difficult to define than disease, it cannot exert a clear moral appeal, or at least not as clear as from disease.

From a value-theoretical point of view, it can be maintained that there are relative differences. Health and disease have different strengths in moral appeal. In particular, it can be argued that disease has more moral weight than health. If we can strengthen the health (in terms of wellbeing) of a healthy person with the exact amount that we can reduce the severity of a disease (in terms of suffering) in a diseased person, one can argue that we should prioritize the latter. This line of thinking can find support in Schopenhauer’s work, where sympathy with others (Mitleid) is the root of ethics and according to whom “[a] thousand pleasures are not worth one pain” [33].

A difference in moral appeal between health and disease in terms of wellbeing and suffering can also be argued for from the purpose of morals [34]. For example, Bernard Gert argues that “evils or harms play a much more important role in morality than goods or benefits. … Normally, promoting goods is not a moral matter at all” [35]. Related to the work of Bernard Gert [35], Héctor Wittwer argues that health is a byproduct of treating disease and that we do not have a duty towards a byproduct, when we have a duty to the product (reducing disease) [36]. Such views can be traced back to the German philosopher Immanuel Kant who counted the duty to help suffering people to be an (imperfect) duty, contrasted to the perfect duty not to kill other persons [37].

An asymmetry between health and disease may also be argued for from various perspectives in virtue ethics. For example, virtues like compassion can be triggered by (the suffering of) disease, but not by the pleasure of health. To some, avoiding pain is considered to be (near) universal end for (human) beings [38]. Thus, disease is something concrete to avoid, while health or betterment are subjective and diverse [39]. Accordingly, other persons’ disease induces reactions such as sympathy and empathy, prompting virtues like compassion providing a moral appeal to help. There are no corresponding reactions to people’s health, it may be argued, and no equivalent virtues aiming to promote person’s health (or if there are, they are not that strong).

Additionally, one may argue for a difference in moral appeal from the logical and conceptual differences between health and disease (as from wellbeing and suffering). In the study of pairs of ethical notions, such as good and bad, health and disease, happiness and suffering, and life and death, the Norwegian philosopher Knut Erik Tranøy found that negative notions have a higher ‘moral weight’ than positive ones. The members of such pairs “are in fact ‘asymmetric’ and that the negative members of such pairs of notions are more fundamental and definite, logically speaking, and operationally more important than the positive members” [11]. “That *X* is in pain is in a sense the only thing *Y* needs to know and to see in order to feel that he ought to help, if he can, to relieve the pain. But suppose *X* is not in pain of any kind. Then even if *Y* thinks he can increase *X*’s pleasures, he cannot know if there is a call on him, if he has the right even, to attempt to do so unless he has criteria beyond his own ability. With regard to pain, *can* implies *ought*. With pleasure, can mere implies *Should I?*” [11].

Moreover, it can be argued that health and disease are different phenomena, and therefore have different moral implications. As mentioned, Gadamer argued that health is (more) enigmatic than disease [32]. As pointed out by Art Caplan, “it is much less difficult to obtain agreement across social classes and different cultures about those states of the mind and body that constitute diseases than it is to secure agreement about which states are to be viewed as healthy” [40]. We recognize exemplars of disease, but not of health [41, 42], and we have taxonomies of diseases, but no classifications of health [43]. Moreover, the epistemology of disease is prior to the epistemology of health [44]. Health and disease are also experienced differently, as disease is an occurrent phenomenon whereas health is dispositional [41]. While disease is felt, health is not (in the same manner) [32]. Health is the simple awareness of living [45]. Moreover, disease is considered to be temporal, while health is atemporal [46]. Hence, disease is a more distinct phenomenon than health, the argument goes, and thus has a clearer moral appeal.

In sum, while classical utilitarianism provides arguments for a symmetry in moral appeal from health and disease, negative utilitarianism and a wide range of other positions support an asymmetry view. Table 3 provides an overview of the arguments.

**Table 3:** Summary of the how wellbeing- and suffering-based conceptions of health and disease support an (a)symmetry view of moral obligation

**Theory, position, perspective**	**Relevant for or applied on health and disease**
Classical utilitarianism	We have an equally strong moral obligation to promote other persons’ health as we have to reduce (avoid or diminish) their disease
Negative utilitarianism	There is a direct moral appeal from disease that is not matched by a moral appeal from health
Ontology: - Substantial differences, different kinds, - Eliminativism	Disease has morally relevant elements or features (such as suffering) that health does not have One of the concepts cannot be defined or operationalized. For example, the enigma of health (Gadamer)
Value theory, axiology: there are relative differences, different strengths	Disease has more moral weight than health
Rule-based ethics/meta-ethics: morals have an asymmetrical purpose	Disease plays a more important moral role than health
Virtue ethics: the moral asymmetry of virtues	Disease evokes virtues, health does not (or less so)
Philosophy of language: logical and conceptual differences	Disease provides a higher moral pressure and has more moral weight than disease as do other pairs of value-laden concepts

## Discussion

While naturalists have a symmetry view on health and disease, they in general hold that neither of the concepts have any moral appeal. Normativists on the other hand agree that both concepts are value laden and have a moral appeal, but they cannot agree on whether the appeal is symmetrical or not. For all parties, it is difficult to demonstrate what it is with health and disease that makes them similar or different (and how) in order to spur a difference in moral appeal. From a pluralist point of view, the arguments for an asymmetry view appear more compelling than the symmetry view. There are many more (and different) arguments for the difference in health and disease being morally relevant for a moral appeal. Without confusing quantity with quality, there are several good reasons to claim that there is a stronger moral appeal from people’s disease than from their health.

It is important to underscore that this is a general claim, as I have not investigated whether there is a difference in a specific type of moral appeal from a particular conception of health and a certain conception of disease. Specific studies of particular kinds of moral appeal (such as moral imperative) from certain conceptions of health and disease are of course needed and welcome but are beyond the scope of this study.

One highly justified objection is that the study addresses disease and not illness (in terms of negative first-person experience) as illness is more directly related to pain and suffering. There are two main reasons for this. First, illness does not have a clear counterpart, as health is much broader than the absence of illness. Second, illness is much broader than what we can expect to be morally responsible for. We cannot be responsible for all people’s negative first-person experiences — especially not in the health care or health policy setting.

**Fig. 2:**
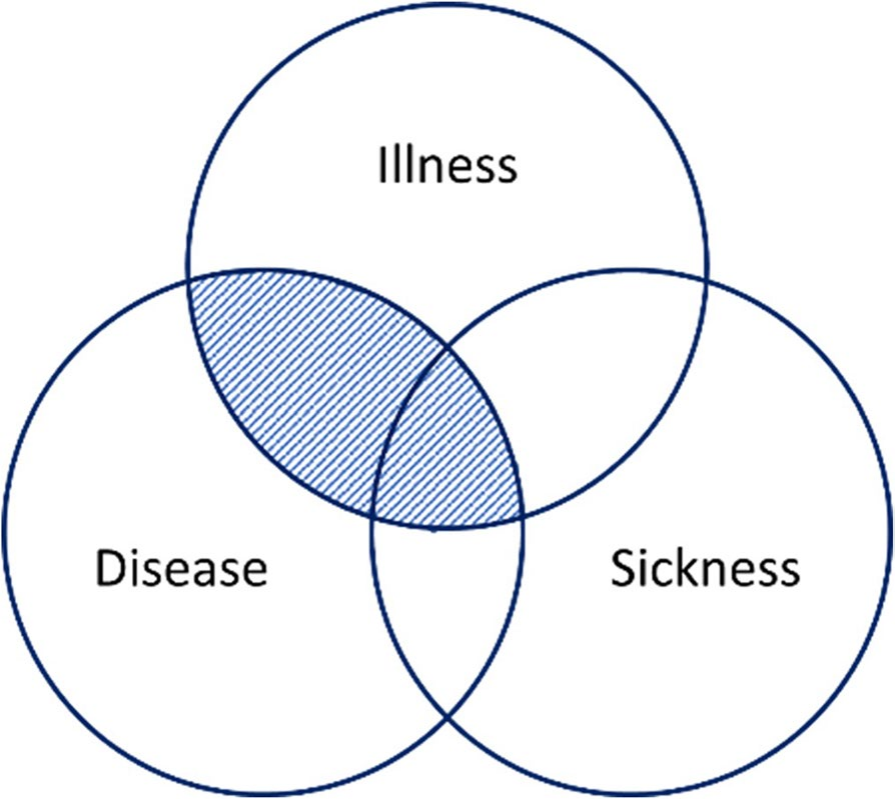
The area of human malady (disease, illness, sickness) that is discussed in this article. See also [48]

In the context of health professionals, healthcare systems, and health policy, we can only be expected to have moral obligations towards those parts of people’s negative first-person experiences that are related to disease. A person may suffer from a wide range of external factors, such as poverty. However, these types of suffering are beyond the subject matter of healthcare. Healthcare can only be responsible for those parts of suffering (and illness) that fall under its subject matter, i.e., where one can provide physical, biochemical, biomolecular, or mental characteristics of the condition that is thought to cause or make up the suffering (disease) and potentially can reduce the suffering. Ought implies can [47]. Figure 2 tries to illustrate the area that is addressed in this study. The reason why the area of disease, but not illness or sickness, is not included, is that it is not directly related to pain or suffering.

Moreover, if one defines health as the absence of disease and conversely, the moral appeal should be symmetrical. However, this ideal model is difficult to defend [42]. While health and disease are interdependent, they are neither mutually exclusive nor exhaustive. You can be both healthy and diseased as well as neither healthy nor diseased. For details, see [42]. Moreover, it may be argued that it is more fruitful to discuss the (a)symmetry of wellbeing and suffering than that of health and disease. However, as we classify and attribute rights to disease and not suffering, it seems warranted to focus on health and disease.

In general, if there is a stronger moral appeal from disease than from health, this has substantial implications, both for patients, professionals, health policy makers, and society at large.

### Implications

First, it has implications for the ethos of medicine, i.e., its values and goals. It means that health professionals, health policy makers, as well as society at large have to give priority to disease demotion over health promotion. Treating persons suffering from malaria or cancer here and now should have priority before screening healthy persons for (vague) indicators of potential future disease and before enhancing the health and wellbeing of people. While radical, the moral primacy of disease is not new for defining the goals of medicine and health care [13, 49, 50].

Second, it specifically implies that there is a stronger personal, professional, and social obligation towards persons with a disease than persons in (good) health. Within a healthcare setting, both treatment and palliation of manifest disease should then have higher priority than disease prevention, which in turn should have higher priority than health promotion. This radically opposes the maxim that “an ounce of prevention is worth a pound of cure” [5, 7].

Third, the asymmetry in moral appeal also has implications for which medical providers (specialties), which patient groups, and which countries/regions deserve more resources and attention. Those patient groups, specialties, or countries/regions with the highest burden of disease should have the most resources.

Fourth, the asymmetry has implications for the enhancement-treatment debate [51], where it implies that the treatment of disease has priority before the enhancement of health. However, first and foremost, it challenges the general assumption of symmetries between wellbeing and suffering as well as between health and disease.

Fifth, healthcare (or more precisely, disease care) should have priority over other services directed at improving people’s health and wellbeing. That is of course not to say that such services should have no priority. So far, nothing has been said about the strength of moral appeal or the extent of asymmetry. This is the topic of one or more separate studies. However, some preliminary notes can be made.

**Fig. 3:**
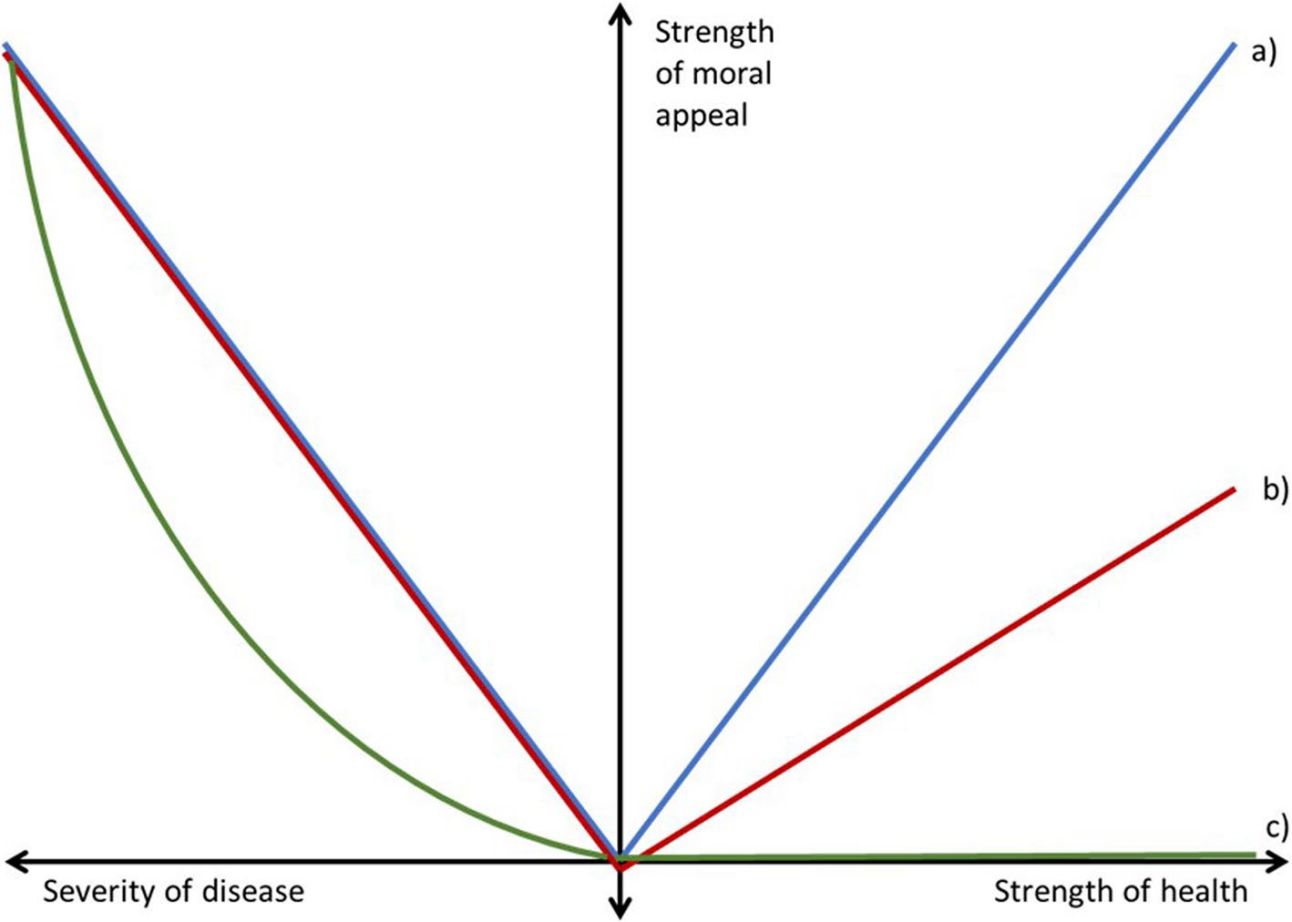
Relationship between severity of disease versus strength of health and the strength of moral appeal. **a** Utilitarian (symmetrical) account, **b** negative utilitarian (asymmetrical) account, **c** one example of an eliminativist account (where health has no moral appeal as it does not exist)

The various theories and positions studied above give different answers to the relationship in the moral appeal from health and disease. Figure 3 illustrates the relationship for some of the discussed perspectives.

### Limitations

There are certainly many limitations to this study.

First, it may be argued that medicine has a wide range of activities beyond the categories of health and disease that then are not covered by this analysis, e.g., vasectomy and tubal ligation. However, health and disease are basic concepts of medicine’s goal related to its basic moral appeal.

Second, the asymmetry in moral appeal could as well be discussed in terms of prioritarianism, where those suffering are worse off than those who are well. However, this is a slightly different study.

Third, as noted at the outset, the study has not scrutinized specific conceptions of health and disease. Only general normativistic and naturalistic conceptions have been investigated. Further and more detailed studies are surely most welcomed.

Classical utilitarians may argue that it is not the number of arguments for an (a)symmetry in moral appeal that counts, but the quality of the argument for symmetry. However, both sides of the (a)symmetry divide have problems in explaining what with health and disease that provides a (difference in) moral appeal. There may of course be differences in how compelling one finds that arguments on both sides.

Fifth, there are certainly many perspectives that have not been addressed. For example one can argue for an (a)symmetry from a rights-based perspective: “disease and disability become the object of concern in Western society because they are seen as a threat to equal opportunity, and in turn to the moral foundation of economic life” [52]. Or from a pragmatic point of view that it is much more resource demanding to improve a person’s health than to reduce a person’s disease. Moreover, various biases may also account for the moral relevance of health and disease, e.g., loss aversion according to which a loss is considered as more negative than an equivalent gain (of wellbeing). 

Sixth, it can be argued that health and disease are constituted by other phenomena than wellbeing and suffering, for example by disability or harm [53–55]. While this is true, wellbeing is widely used to define the concept of health as an expressed goal of healthcare [56–59]. The same goes for suffering [60–65].

Seventh, it may be argued that suffering and wellbeing can be defined and measured in many ways [66–69], that they are implicit negations, or that suffering is not bad or wellbeing or happiness is not good [70]. This is clearly true, and as pointed out, it must be studied in relation to explicit conceptions of health and disease.

Eight, this study has not differentiated between different categories of moral appeal, for example between types of moral actions and obligations, such as (a) *perfect obligations*, which require compliance without exception; (b) *imperfect obligations*, which allow for discretion with respect to their fulfillment; and (c) *supererogatory actions*, which are very highly regarded from a moral point of view but not morally required [71, 72]. Again, this needs much more elaboration than allowed within the scope of this study.

Ninth, due to limited space and scope the study has not taken important temporal aspects into account. For example, consequentialism (and other presented positions) have developed over time which can provide important nuances. However, I have referred to “classical consequentialism” and other specific positions. More detailed elaborations on the development of the various positions are warranted and welcomed. Moreover, the important trade-off between disease treatment here and now having consequences in the far future versus health-promoting measures having consequences in the near future [73] have not been addressed either, as this warrants a separate study. Such a study would need to take into account both present and future (potential) harms and benefits related to present and future resource allocation related both to health and disease.

## Conclusions

To address the question of whether there is a stronger moral appeal from people’s disease than from their health, I have investigated health and disease in terms of naturalist and normativst conceptions of the concepts. While naturalist conceptions of health and disease are mute on moral appeal there are many normativist arguments for an asymmetry between health and disease that provide substantial support for a stronger moral appeal from disease than from health. This has a wide range of implications, for patients, professionals, health policy makers, and for society, especially with respect to priority setting. In particular, treatment, palliation, and prevention of disease should have priority to the promotion and enhancement of health.

## Data Availability

All data are available in the article.

## Abbreviations

GDP: Gross Domestic Product
OECD: Organisation for Economic Co-operation and Development
WHO: World Health Organization
